# Combating challenges in CAR-T cells with engineering immunology

**DOI:** 10.3389/fcell.2022.969020

**Published:** 2022-10-10

**Authors:** Clement Yisai Wang, Stephanie Po Ting Cheung, Ryohichi Sugimura

**Affiliations:** School of Biomedical Sciences, LKS Faculty of Medicine, The University of Hong Kong, Hong Kong, Hong Kong SAR, China

**Keywords:** car-t, antigen, immune checkpoint inhibitors, cancer immune cell therapy, tumor micro environment

## Abstract

Chimeric antigen receptors (CAR) T cells (CAR-T) mark a significant step towards producing safe and effective personal anticancer treatments. CAR-T strategies engineers the T cells from the patients to allow specific binding to a tumour-specific antigen. CAR-Ts are a second-wave offensive strategy to clear out remaining chemotherapy-resistant tumour cells. Though showing practical antitumor abilities in multiple haematological malignancies and solid tumour cancers, the issues of antigen escape, tumour infiltration/penetration, and toxicity side effects limit the usage of prolonged CAR-T therapies. However, engineering immunology has exploited human stem cell-based CAR-T therapies and the development of CAR-M (macrophage) therapies to combat the disadvantages of conventional CAR-T therapies. In this review, we will highlight the challenges of CAR-T therapies and combat them with engineering immunology for cancer immunotherapy.

## Introduction

Chimeric antigen receptor (CAR) T cells therapies are one of the most exciting developments in recent cancer immunotherapies. CAR-T cells act as engineered immune cells that track down a targeted antigen, then induce apoptosis on the cells bearing the targeted antigen. Collectively, CAR-T stimulated responses are independent from MCH surface receptor induced immune response. Though there are many clinical trials undergoing FDA and clinical approval currently, there are many challenges that CAR-T immunotherapies still face such as antigen escape and cytokine release syndrome, discussed later in this review. Current research are also focusing on implementing CAR constructs on other immune cells such as macrophages and improving the safety profile of current CAR-T cells. In this review, we review the current progress of CAR-T immunotherapy and some recent clinical trials, as well as challenges of CAR-T immunotherapy and alternative solutions.

## The chimeric antigen receptor construct

The CAR construct was genetically modified and plated to thousands of patients’ T cells in the laboratory by adding a gene for an artificial CAR protein. This manufacturing retains the specificity of the antibody immune system and enables T cells to identify and latch onto a specific antigen on the malignant cells and destroy them. The CAR comprises three domains, an extracellular antigen recognition domain, a transmembrane spacer domain, and an intracellular domain (Guedan et al., 2019; Sadelain et al., 2013). Current CAR designs are dedicated to discovering scFvs that recognize malignant cells with high efficiency and low toxicity to normal cells. By incorporating additional signalling domains, the CARs have evolved to the fourth generation (Han et al., 2021; Kalos and June, 2013).

**FIGURE 1:**
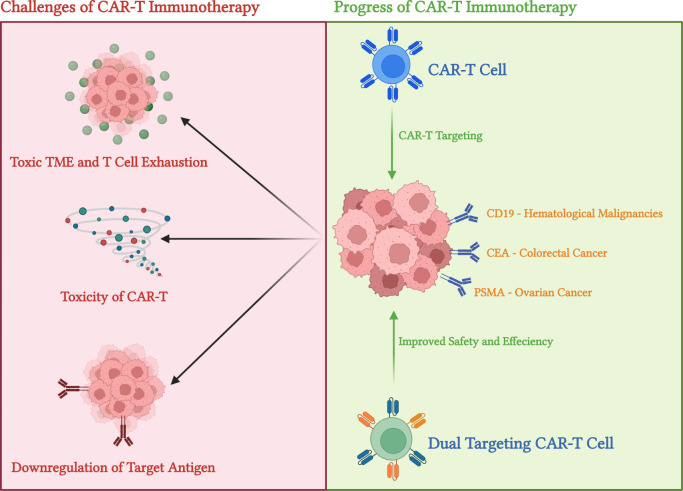
Evolution of CAR constructs. This diagram displayed the first through fourth generations of CAR constructs attached to modified T cells. The diagrams display the compartments, antigen-binding site, and intracellular signalling domain and its components on either generation of CAR constructs. scFvs are marked red and green with the linker and hinge protein in purple. In addition, costimulatory domains inside the CAR-attached cells are combined with the CD3ζ domain. The latest generation of CAR constructs produces molecules to modify DNA transcription and cytokine release. Diagram produced with BioRender

The affinity and avidity of the extracellular antigen-binding region, single-chain variable fragment (scFv), of the CAR are derived from the Fab of the monoclonal antibodies, which are the variable light (VL) and heavy (VH) chains (Jayaraman et al., 2020). Though scFCs are not the only extracellular chains available. VL and VH are incorporated into the scFv of CAR as the extracellular antigen-binding domain. When scFv of CARs binds to the antigen CD19, the CAR T cells are activated with the amplified activation signals in a cascade manner to proliferate and kill the tumour cells. However, CAR-T cell therapy has side effects and other rare but serious adverse events associated with acute toxicities, cytokine release syndrome (CRS), and neurotoxicity (Sterner and Sterner, 2021). The spacer is the transmembrane region that links the extracellular scFv and the cytoplasmic Fc of the CAR construct. Hinge is the domain between the scFv and the cell membrane derived from CD8, CD28, IgG1, and IgG4 (Qin et al., 2021). A study found that CD19-CAR T cells with IgG4 CH2-CH3 hinges were functional *in vitro* and helped boost CAR T cells’ expansion and antitumor efficacy (Abraham and Stenger, 2013) (Hung et al., 2018). There are four generations of CAR T cell types based on different moieties. The first generation consists only of the signalling transduction domains, the CD3ζ chain. The second generation involves additional costimulatory domains, such as CD28, 4-1BB, CD27, MYD88, or OX40, which are all based on the first generation. With a fixed CD28, another supplemental costimulatory domain, and CD3ζ, these components make up the third generation of intracellular signalling domains. The fourth generation with an extranuclear factor of activated T-cells (NFAT) is based on the second generation of CAR constructs (Figure 1) (Green et al., 2018) (Guedan et al., 2019). For regular T cells, multiple antibodies, including TCR, CD8, CD4, CD45, CD3, and costimulatory molecules, like CD28, are synchronized to activate T cells fully. As a result, the costimulatory and signal transduction domains are the key regulators of auto-immune diseases (Abraham and Stenger, 2013). Phosphorylated immunoreceptor tyrosine-based activation motifs (ITAMs) of the TCR and CD3 components promote zap-70 activation and follow a signalling cascade to activate T cells (Green et al., 2018).

## Chimeric antigen receptors T cell immunotherapy usage in specific cancer conditions

Since the introduction of CAR-T immunotherapy in human clinical trials in 2017 (MT 2017-45), there have been studies focused on the effectiveness of CAR-T therapy on a variety of haematological malignancies and solid tumours. This section will discuss CAR-T efficiency in a few cancers that have been proven to be most significant or provided the most significant clinical data.

### In hematological malignancies

CAR-T immunotherapy has demonstrated remarkable effects in numerous haematological malignancies, including acute lymphoblastic leukaemia (ALL), chronic lymphoblastic leukaemia (CLL), lymphomas, as well as multiple myeloma (MM). In ALL patients, thanks to Kymriah developed by Novartis, the CD19-targeting CAR-T therapy showed significant antitumor effects when initially treated (Sun et al., 2018). However, long-term exposure to CAR-T cells causes antigen escape in most patients (Hoyos et al., 2010; Sommermeyer et al., 2017). Both CD20 and CD22 have been proven to be potential candidates for CAR construct targeting, CD20 showed promising results for initial CD19-replacement treatments, but CD22 showed more outstanding results for relapsed patients who were initially treated with CD19 CAR-T immunotherapy (King et al., 2019). Thus, more focus has been on CD22 targeting CAR-T therapies, and two main anti-CD22 agents are under research. CD123 is another novel pathway currently under research; mice model studies have shown great antitumor efficiency (Pemmaraju, 2017). In CLL, the malignant results in immunodeficiencies in patients and produces more complicated symptoms than ALL. Though the introduction of CAR-T treatments in CLL is relatively recent due to complications of CLL, compared to traditional stem-cell transplants with chemo- and radiotherapy, CD19 CAR-T treatments showed some curative effects with limited T cell expansion and proliferation due to immunodeficiency in patients (Turtle et al., 2016). Lymphomas, including non-Hodgkin lymphoma (NHL), Hodgkin lymphoma (HL), anaplastic large cell lymphoma (ALCL), and follicular lymphomas (FL), are all traditionally treated with chemotherapy and monoclonal antibody treatments. Though successful, several patients still suffer from disease determination due to complicated disease progression (Zhu et al., 2015). With the recent introduction of anti-CD19 CAR-T therapy, 75 percent of patients in the trial showed at least partial remission (Lin et al., 2019). In B cell lymphomas, an overwhelming expression of CD20 in malignant cells provokes the development of anti-CD20 CAR-T therapies (Kochenderfer et al., 2010). In a trial completed by Wang et al. (2014) anti-CD20 CAR-T showed an effective response and delay to toxicity from advanced diffuse large B-cell lymphoma. Like CLL, multiple myeloma (MM) causes anaemia, immunosuppression, hypercalcemia, bone lesions, and renal failure due to abnormal plasma cell aggregation caused by plasmacytoma (Ramos et al., 2016). Anti-CD19 CAR-T demonstrated limited success due to the lack of expression of CD19 in MM patients (Hosen, 2019). Though there are many treatment options for haematological malignancies, such as chemotherapy and hematopoietic stem cell transplantation (HSCT), CAR-T therapy represents one of the most controlled therapies in terms of antigen targeting. With great potential to make CAR-T safer and accessible for more patients, CAR-T therapies are slowly overtaking the use of traditional therapies.

### In solid tumors

Unlike blood cancers, where cancer cells express special or unique surface protein markers, solid tumour cells normally do not express one tumour-specific marker (Sterner and Sterner, 2021). Instead, in solid tumours, various tumour-associated antigens are upregulated in cancer cells. However, these antigens are also naturally expressed on a lower level in the human body (Qin et al., 2021).

In ovarian cancer, there is a high level of tumour recurrence even after surgery and chemotherapy procedures, leading to an increased demand for alternative solutions. Tumour-associated glycoprotein 72 (TAG72) is highly expressed on the cancer cell surface, leading to research on anti-TAG 72 CAR-T cells (Cao et al., 2020; Murad et al., 2018). Another option for treating ovarian cancer is using HER2 targeting CAR-T cells. The HER2 targeting CAR-T cells demonstrated high capabilities to suppress the growth of ovarian cancer cell line SKOV3 (King et al., 2019). All three CAR constructs are desirable at this stage, and further testing will be needed to determine the superior one. Unlike ovarian cancer, where selecting an antigen to target is somewhat complicated, antigen selections in prostate and renal cancers are far simpler. In prostate cancer, the prostate stem cell antigen (PSCA) and prostate-specific membrane antigens (PSMA) are used for CAR construct targeting (Slovin et al., 2013). PSMA targeting CAR-T cells show great reactivity to human prostate cancer cells and cytotoxic ability (Hillerdal and Essand, 2015; Weimin et al., 2020).

In brain cancers, the most common type of primary malignant brain tumors is glioblastoma (GBM). Current available treatment is tumour removal surgery followed by chemotherapy, which yields the 2 years survival rate of patients below 30 percent (Ostrom et al., 2018). Currently, CAR-T immunotherapy targeting IL13Ra2 are being research as the target is a commonly expressed membrane-bound protein in over three-quarters of GBMs and is associated with activating the mTOR (mammalian target of rapamycin) pathway, which favours tumour growth (Thaci et al., 2014; Filley et al., 2017). Another target considered for GBM targeting is the human epidermal growth factor receptor 2 (HER2) (Klampatsa et al., 2020). HER2 is usually overexpressed in GBM cells and antigen escape of HER2 targeting CAR-T cells are usually lower. Both targets demonstrated improved quality of life and tumour lysis abilities in patients up to 7.5 months (Shen et al., 2019). Compared to the current available treatments, it is a great improvement in treatments as the expected GBM patient survival of CAR-T treatments is over 30 percent (Ostrom et al., 2018) There are clinical trials regaring both mTOR and HER2 targeting CAR-T cells (NCT02208362 and NCT02442297 respectively) and it is aimed to introduce commercially available CAR-T immunotherapies for GBM patients while also trying to overcome some challenges of CAR-T cells (described later in this review).

The use of CAR-T immunotherapies in colorectal cancers are heavily investigated. The carcinoembryonic antigen (CEA) is a common target for current CAR-T immunotherapies as the surface marker expressed on over 80 percent of colorectal cancers (Katz et al., 2015). Intravenous injection of CEA in humanized mice models displayed significant tumour lysis ability and reduction in tumour growth over a period of 3 months. Compared to previously used TM4SF1 or EpCAM targeting CAR-Ts, the anti-CEA CAR-Ts displayed greater anti-tumour abilities as wells as reduction in antigen escape effects (Riccardo et al., 2018). However, in all the CAR-T immunotherapy, the safety profile should be improved (Katz et al., 2015).

## Chimeric antigen receptor T cell immunotherapy clinical trials

With growing interest in producing CAR-T immunotherapy products for the general public, an increase in CAR-T-related clinical trials has been initiated. In this section, we will briefly discuss some clinical trials as examples.

In a clinical trial regarding haematological malignancies performed by Frey and Porter (2016), injection of anti-CD19 CAR-T achieved complete remission. However, those CAR-T treatments are always accompanied by treatment-related toxicity and side effects (discussed in detail in the next section) (June et al., 2018). In lymphomas, single antigen targeting CD19 therapy may be limited. Thus, antigen replacement is currently under study. There are two candidates currently being considered to replace CD19 targeting (Zhang et al., 2019a). The first one is CD269 (BCMA), which is highly expressed in mature B and plasma cells (Carpenter et al., 2013; Roex et al., 2020). The second target is CD138, which is highly expressed in malignant plasma cells. Clinical trials of CD138 (NCT03672318) showed CAR-T cell migration to bone marrow and high antitumor efficiency without significant toxic side effects (Han et al., 2021). In solid tumours, ovarian cancers, in particular, a solution is needed for the antigen escape challenges of CAR-T therapies. Reports using humanized mouse models claimed cytotoxic effects and cytokine production once anti-TAG 72 CAR-T was injected, but proliferation and toxicity-related issues were pursued heavily. Other clinical trials have revealed MUC16 as a potential target candidate (NCT02498912). Through IV or intraperitoneal injection, MUC16 CAR-T demonstrated capabilities of slowing ovarian cancer progression and achieved complete remission in some mouse models (Chekmasova et al., 2010). The most recent clinical trials by Junghans et al. and Slovin et al. demonstrated high efficiency and impressive safety profile in renal cancers (NCT05354375) (Junghans et al., 2016; Klampatsa et al., 2020). In renal cancers, carboxy-anhydrase-IX (CA-IX) is the main target of CAR-T cells (Liu et al., 2021). Though CA-IX is expressed on normal tissue cells in the small intestine and duodenum, CA-IX is a critical antigen for renal cell carcinoma, and further hypoxia due to carcinoma drive upregulation of CA-IX. In addition, CA-IX CAR-T cells showed the capabilities of participating in cytokine production and tumour suppression (Pantuck et al., 2008; Tafreshi et al., 2014). A list of previously discussed CAR-T related clinical trials are presented below in Table 1.

**TABLE 1:** Information of CAR-T Immunotherapy clinical trial mentioned.

Lead investigators and date	Clinical trials reference number	Cancer type	Target
Frey et al. (2020)	NCT01029366	Haematological Malignancies	CD19
Tuchman (2018)	NCT03672318	B Cell Lymphoma	CD138
Chekmasova et al. (2010)	NCT02498912	Ovarian cancer	MUC16
Murad et al. (2018)	NCT05354375	Renal cancer	CA-IX
Badie (2014)	NCT02208362	GBM	mTOR
Ahmed (2015)	NCT02442297	GBM	HER2

The above table states relevant information, including clinical trial reference numbers, for all the mentioned clinical trials mentioned in this review. The target antigen and its use in different cancers are also stated.

## Challenges of chimeric antigen receptor T cell immunotherapy

Though CAR-T immunotherapy has many advantages compared to traditional chemotherapy and surgery, CAR-T technologies are still under heavy advancement due to some challenges it faces. In this section, we will briefly discuss CAR-T immunotherapy’s limitations from the angle of antigen escape, issues with infiltration, CAR-T toxicity challenges, and lastly, some current modifications to improve the CAR-T safety profile.

### Antigen escape

**FIGURE 2:**
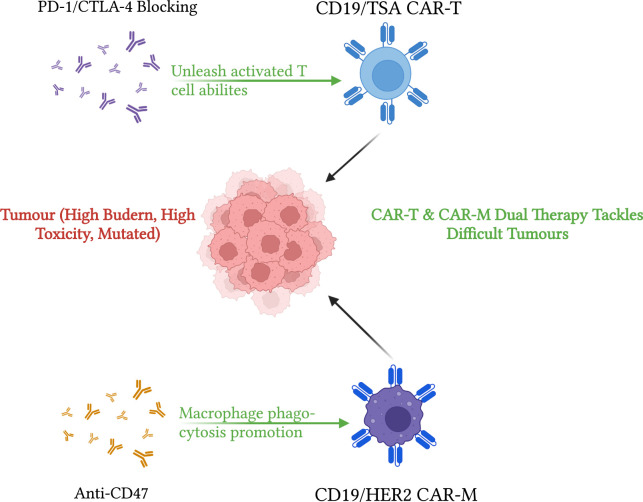
Summary of CAR-T in action and limitation of CAR-T immunotherapies. This diagram summarizes the main limitations and usages of CAR-T immunotherapies. The top part indicated the three main limitations of CAR-T cells: antigen escape—or downregulation of target antigen upon CAR-T activation, limited tumour infiltration due to toxic tumour microenvironment (TME), and T cell exhaustion as toxicity side effects of CAR-T. On the other hand, using CD-19 targeting (for haematological malignancies) and glycoprotein or tumour-associated Antigone (TAA) targeting (for solid tumours), CAR-T cells demonstrate excellent antitumor effects in clinical conditions. On top of that, dual CAR constructs and Tandem CAR constructs methods may be used to aid antigen escape effects. Diagram produced with BioRender.

One of the most common observations during the later period of CAR-T immunotherapy treatments is tumours developing resistance to single antigen targeting constructs. The phenomenon, called antigen escape, is seen in later stages of treatment as tumour cells lose particle or complete expression of target antigens. In the case of targeting CD19 in acute lymphoblastic leukaemia, 30–70 percent of patients showed downregulation or loss of target CD19 expression after treatment (Liu et al., 2021; Maude et al., 2015). Similarly, downregulation or loss of BCMR is observed in multiple myeloma patients treated with prolonged BCMR targeted CAR-T immunotherapy (Gagelmann et al., 2019). Antigen escape is also seen in solid tumours, as shown in IL13Ra2 targeting CAR-T strategies for glioblastoma by Brown et al. (2016) tumour IL13Ra2 recurrence decreased significantly as treatment progressed (Figure 2). Multiple antigen targeting strategies are now highly valuable as it reduces the effect of single-antigen escapes in haematological malignancies and solid tumours. Multiple antigens targeting CAR constructs use either dual CAR constructs or tandem ones that are singular constructs with two single-chain variable fragments (Rafiq et al., 2020). Reports from current dual targeting strategies (CD19/CD22 or CD19/BCMA) have shown promising results (Wolchok et al., 2017; Klichinsky et al., 2020). Preliminary clinical trial results of CD19/CD22 dual targeting CAR-T constructs showed efficiency in adult patients with acute lymphocytic leukaemia and diffused large B cell lymphoma (NCT00924326) (Hossain et al., 2018; Dai et al., 2019). Furthermore, CD19/BCMA dual CAR therapy showed high efficiency against malignant cells and improved safety profile (Stijn et al., 2018).

### Chimeric antigen receptor T cell tumor infiltrations

Solid tumours are more effective at using toxic tumour microenvironment and physical barriers to block interaction from immune cells. While tumour facilitating cells, such as myeloid-derived suppressor cells (MDSCs), tumour-associated macrophages (TAMs), and regulatory T cells (Tregs), can migrate into the tumour microenvironment, many immune cells cannot (Sterner and Sterner, 2021). On top of that, PD-1 and CTLA-4 downregulation can decrease antitumor immunity further (Snyder et al., 2014). Immunosuppression and physical tumour barriers are two main factors affecting CAR-T effectiveness (Figure 2). Under a heavy immunosuppressive environment, the lack of T cell expansion and persistence of Tregs due to T cell exhaustion results in poor or minimal CAR-T responses. Thus, CAR-T therapies are often combined with immune-checkpoint blockade therapy to provide the two essential components of the CAR-T antitumor effect: CAR-T is capable of penetrating and infiltrating solid tumours, and blockade therapy, such as PD-1/PD-L1 blocking therapy, of providing persistent T cells and its expansion (Eagar et al., 2002). In pediatric patients with B-ALL, CAR-T and PD/PD-L1 blocking strategy combination therapy improved CAR-T efficiency and its antitumoural effects (Sterner and Sterner, 2021). There are currently many studies in solid tumours examining the antitumor efficiency of combination treatments for a variety of cancers. However, it is harder to gauge the effects of combination therapies due to more toxic environments generated by solid tumours. Recently, more researchers have been focusing on engineering CAR constructs to resist hostile TMEs caused by TGF-β (Quail and Joyce, 2013). Another strategy is to engineer CAR constructs to carry immunostimulation cytokines to aid the host immune system and improve antitumor effects (Rafiq et al., 2020).

### “On target, off TumVor” effects

The main goal of selecting a target antigen to ensure the therapeutic effects of CAR-T immunotherapy is to limit the “off-target” effect. Solid tumour express antigens are sometimes also expressed in normal tissue cells. Thus, the CAR-T antigen must maximize therapeutic effects while minimizing other harmful effects. One current method to overcome this issue is to target tumour-specific post-translational modifications such as truncated O-glycans (Steentoft et al., 2018). Four major targets are B7-H3, TAG72, MUC1, and MUC16 (Alena et al., 2010; Koneru et al., 2015; Du et al., 2018). In particular, first generations of TAG72 CAR-T contrasts prove to have no antitumor effects, but newer TAG72 targeting therapies using regional delivery have proven some effectiveness against solid tumours (Lin et al., 2019). Other targeting methods, such as B7-H3 targeting CAR-T constructs, demonstrated anti-tumoral effects when murine PDAC tumour models expressed PD-1 (Du et al., 2018). Though CAR-T immunotherapy has already proven to be a revolutionary cancer treatment method, high mortality in treatment groups due to CAR-T-related toxicities prevents CAR-T treatments from being the first-choice treatment for any cancer. The toxic side effect of CAR-T is due to several factors innate to the construct or the target of the CAR-T construct and tumour type. Common symptoms of toxicity include cytokine release syndrome (CRS), hemophagocytic lymphohistiocytosis (HLH), macrophage activation syndrome-like manifestations (MAS), and immune effector cell-associated neurotoxicity syndrome (ICANS) (Salter et al., 2018; Rafiq et al., 2020). Frey and Porter’s team found out that in acute lymphoblastic leukaemia/lymphoma (ALA, LBL) patients, CRS is observed among all patients participating in CAR-T treatment to some extent. Additionally, 23%–46% of the patients displayed severe cytokine production dysregulation and *in-vivo* T cell expansion, a sign of CAR-T-related toxicity (Figure 2) (Frey and Porter, 2016).

### Altering chimeric antigen receptor constructs for better safety profiles

Continuous measures have been taken to improve the safety profile of CAR-T therapy. In CD19 targeting CAR constructs, modifications on the CD8 transmembrane protein receptor lowered cytokine release and decreased CAR-T proliferation. With six out of the eleven patients achieving complete remission and no patients reaching level 1 of CRS or ICANS from CD8 modified CD19-CAR-T targeting therapy, optimizing transmembrane hinges and regions is proving to be a useful approach (Ying et al., 2019). Modification of the costimulatory domain is another method to improve CAR construct safe profiles. The costimulatory domain can be modified specifically for different tumour types, burdens, antigen density, and TME. For example, the 4-1BB domain is often targeted to reduce cytotoxicity and improve T cell endurance, reducing T cell exhaustion. While compared to the CD28 costimulation domain, the 4-1BB domain maintains less cytotoxicity, improves CAR-T proliferation, and improves efficiency when the tumour burden is high (Sommermeyer et al., 2017). However, the CD28 costimulatory domain may be needed to achieve high T cell activation rates during tumour infiltration (Sterner and Sterner, 2021).

## Overcoming challenges of chimeric antigen receptor T cells by engineering immunology

### Improving Chimeric antigen receptor T cell therapies with alternate chimeric antigen receptor immune cells

**FIGURE 3:**
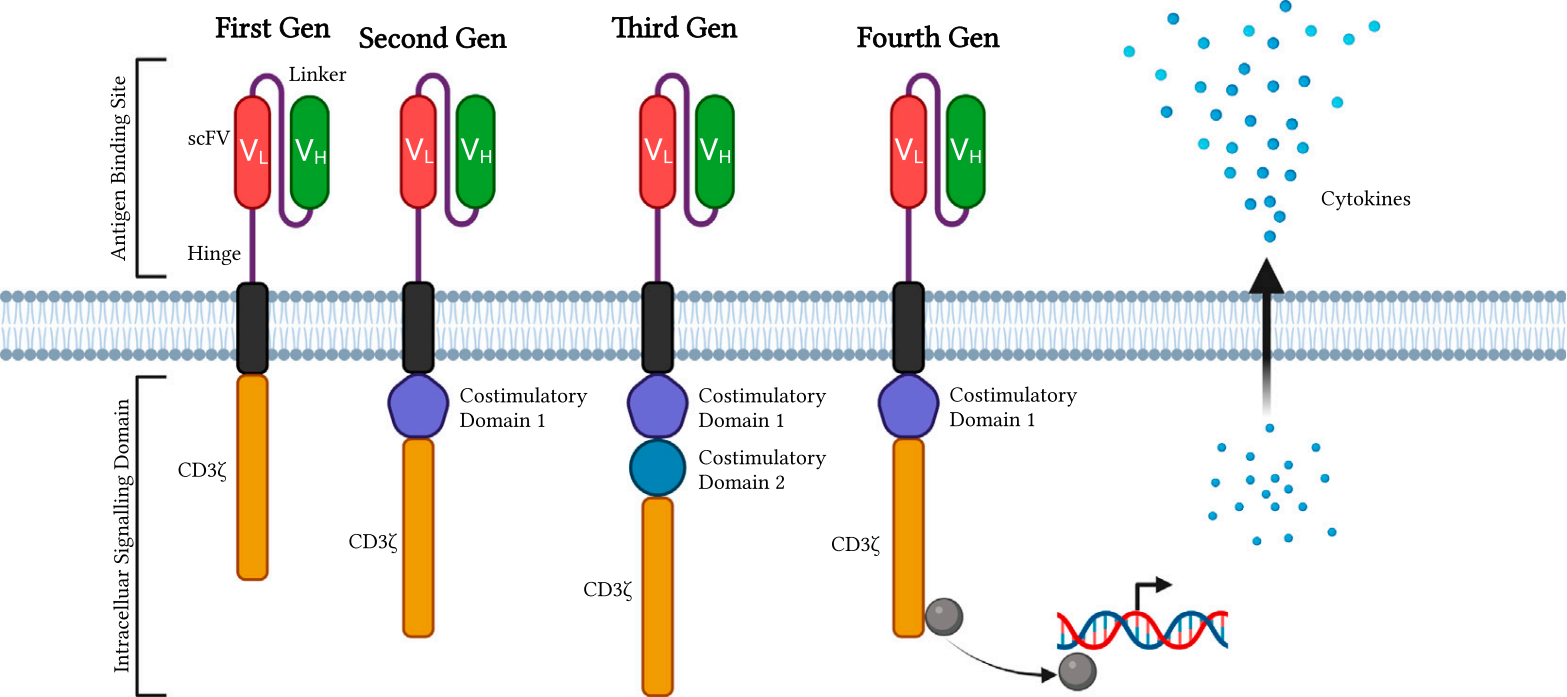
Visualization of CAR-T and CAR-M immunotherapy strategies. This diagram demonstrates strategies for enhancing CAR-T and CAR-M immunotherapies to treat cancers. CD19 or tumour-specific antigen targeting CAR-T can be improved by using PD-1 or CTLA-4 blocking strategies. Specifically, CTLA-4 helps with immature T cell priming after CAR-T injection, which helps improve overall CAR-T efficiency. PD-1/PD-L1 blocking unleashes activated T cell abilities, reduces T cell exhaustion and aids TME toxicity effects. On the other hand, CD19/HER2 dual CAR-M with anti-CD47 treatments have increased phagocytosis activities compared to normal macrophages or CAR-M without CD47 blocking. Both CAR-T and CAR-M contribute to greater tumour lysis abilities. Diagram produced by BioRender.

The PD-1 expression is an important switch mechanism for the adaptive immune system. The expression of PD-1 on tumour-infiltrating lymphocytes is usually associated with poor effector function and cytotoxic efficiency against tumour cells (Maute et al., 2015). In much research currently underway, a combination of CAR constructs and PD-1 blockade is employed to maximize the effect of CAR-T cells in solid tumours (Shadbad et al., 2022). As CAR-T relies on T cell proliferation and cytokine production when invading tumours, any strategies aiding CAR-T action are highly appreciated. The first CAR constructs tested in many solid tumours is used CD19 CAR-T. However, as mentioned previously, solid tumours do not have one unique defining tumour-associated marker. Thus, designing an effective CAR-T construct to target solid tumours is generally harder. Tumour-associated macrophages, TAMs, on the other hand, are the most abundant cell types within a tumour, taking up around 50 per cent of all cell mass in a tumour. TAMs have antitumor influence over the majority of the tumour growth cycle. Utilizing a similar CAR construct on TAMs, the first generation of CAR-Ms used similar targeting strategies, such as CD19 and HER2 dual targeting (Morrissey et al., 2018). Applying constructs enhanced macrophage phagocytosis abilities while still penetrating tumour barriers in solid tumours. Though promising results, the second generation of CAR-Ms is under development, hoping to gain additional beneficial functions for CAR-Ms such as T cell activation, overcoming plasticity of TAM, and the ability to expand *in vitro*. With those needs, Klichinsky et al. (2020) produced the anti-HER2 CAR-M (CT-0508) while satisfying the requirements of the second generation CAR-Ms, also getting FDA approval after clinical trials with metastatic tumour patients showing great promise of CAR-Ms. Later, Zhang et al. (2019b) improved CAR-Ms to produce from iPSCs by introducing CAR construct by lentiviral transduction and differentiation protocol to myeloid or macrophage lineage. Combining with information from Sage et al., using CD47 blocking strategies and other immune-checkpoint blocking strategies such as PD-1/PD-L1, the antitumor efficiency of CAR-M, and macrophages in general, is improved (Figure 3) (Tu et al., 2021). Compared to CAR-T cells, CAR-M cells’ safety profile is superior in mice models, and it is comparably easier to culture macrophages than mature T cells from human stem cells (Klichinsky et al., 2020). With those two advantages, CAR-M is a promising alternative for further immuno-engineering to produce safe and effective off-the-shelf CAR products.

Alternatively, NKT cells are also emerging and proving their worth in future cancer immunotherapy. Though it exists in only small quantities in the human body, NKT cells, and invariant NKTs (iNKTs) are essential to bridging the gap between the adaptive and innate immune systems. Recently, Heczley et al. created GD2 ganglioside CAR-NKTs targeting neuroblastoma cells. The authors articulated that the generated anti-GD2 CAR-NKTs are toxic to GD2-expressing neuroblastoma cells as well as PD-1-expressing macrophages. On top of that, the authors found that the CAR-NKT efficiency is highly dependent on costimulatory domains such as, as mentioned previously, CD28 and 4-1BB domains. Unsurprisingly, the constructs with both costimulatory domains exhibit the best antitumour effects and longest persistence. Later, *in vitro* expanded antigen-induced NKTs accumulate CD62L + cells. Selective enrichment of more CD62L + cells resulted in cellular phenotypes that surpass previous cytokine profiles and proliferative capacities (Rafiq et al., 2020). Though many projects are still within preclinical trial stages, CAR-NKTs are introducing themselves are the newest member as an alternative to CAR-T therapies and CAR immune engineering products.

### Immune-checkpoint blockade combination therapy

As mentioned a few times before, immune checkpoints are essential to enhance immune cells’ ability to destroy cancer (Ribas and Wolchok, 2018). The two most common targeted pathways are the PD-1 and CTLA-4 pathways. Mechanically different, PD-1 inhibits T cell-mediated cell lysis, and CTLA-4 controls T cell priming processes (Rodriguez-Garcia et al., 2020). *Via* artificial modification of CTLA-4 pathways, CTLA-4 blocking cannot single-handedly break TME and aid T cell efficiency significantly, as suggested by Eager’s team (Du et al., 2018). However, when anti-CTLA-4 is administered with a high dose of the target antigen, CTLA-4 blocking complemented T cell antitumor efficiencies (Snyder et al., 2014). These findings suggest that CTLA-4 blocking causes long-term clinical effects when tumour burden and mutations are high in a patient, as it does not limit T cell priming to a single tumour-associated antigen. On the other hand, PD-1/PD-L1 unleashes further T cell potential from existing tumour-specific primed T cells. Combining CAR-T with PD-1/PD-L1 blocking therapy has already increased CAR-T lysis ability (Zappasodi et al., 2018). It perhaps is possible to see an all-around increase in immune activities after administration of CAR-T therapy, PD-1/PD-L1 blocking, and CTLA-4 blocking (Figure 3). However, the blocking strategies may only work in the unmutated tumour as it is easier for current versions of CAR-T to target. Furthermore, increasing the immune activity to one specific antigen may lead to weakness in other areas, and a simple bacterial or viral infection may prove to be fatal. Thus, further testing and trials are needed to conclude the effectiveness of combing CAR-T immunotherapy and both blocking strategies. On the other hand, with the emergence of CAR-Ms, CD48 blocking immunotherapy is gaining importance in improving macrophage-mediated antitumor immunity. In small-cell lung carcinoma, CD47 blocking targets various targets, including CD56 (neural cell adhesion molecule, NCAM), and promotes significant improvement *via* macrophage-initiated tumour immunities (Weiskopf et al., 2016).

### Future prospects

Improving CAR constructs and its related CAR-T or CAR-M cancer immunotherapies has been an important focus over the last decades. CAR-T therapies have achieved impressive clinical results while the CAR-M field is moving rapidly forward. CAR-T immunotherapy is a very good starting point for the research of future CAR-related therapies. Perhaps when CAR-T therapies are safer and wider in use, they can be used as an initial treatment for naive and early-stage tumours that are easier to target by CAR-Ts or as a last-ditch resort to target as much tumour tissue as possible. Either way, CAR-T therapies, for now, should not be considered a prolonged treatment for patients as there are still many challenges we are facing. The CAR-T and CAR-M fields have complementary effects and disadvantages, further research should focus on minimizing the side effects of each immunotherapy while maximizing the strengths. Other recent reviews on CAR-T therapeutics such as Han et al., 2021 also discuss the therapeutics of CAR-T in haematological malignancies. Their findings are similar to ones presented in this article, an increase in CAR-T treatments in different blood cancers though with many complications. As Sterner also presented, there are many complications regarding CAR-T therapy in general. However, there are potential to expand CAR-T therapy to a wider range of cancers. Looking for alternate or complementing strategies to improve CAR construct efficiencies to replace current traditional cancer therapies such as chemotherapies and invasive surgeries. When effective CAR-mediated immunity is established, we can look forward to integrating with other fields of genomics, transcriptomics, and proteomics to obtain early diagnostics of cancers and effective treatments to avoid prolonged treatments and improve patient satisfaction.
